# The relationship between physical appearance perfectionism on subthreshold depression in college students: the role of gender and fear of negative evaluation

**DOI:** 10.3389/fpubh.2025.1559815

**Published:** 2025-03-26

**Authors:** Yiru Wei, Hong Chen, Bo Sun, Lingli Kong

**Affiliations:** ^1^Applied Psychology, Binzhou Medical University, Yantai, Shandong, China; ^2^Geriatric Psychiatry Qingdao Mental Health Center, Qingdao, China

**Keywords:** physical appearance perfectionism, subthreshold depression, fear of negative evaluation, college student, social pressure

## Abstract

**Introduction:**

Over the past decades, subthreshold depression has emerged as a significant mental health concern among college students, with prevalence rates reaching 40.8%. Despite its substantial impact on psychological well-being, subthreshold depression often remains overlooked due to its failure to meet clinical diagnostic criteria for major depressive disorder. Moreover, the underlying mechanisms of subthreshold depression remain poorly understood. This study investigates the interplay between appearance perfectionism, fear of negative evaluation, and gender in relation to subthreshold depression among college students.

**Methodology:**

Via cross-sectional design, we recruited 820 college students (Mage = 20.78, SD = 2.04) through convenience sampling. Participants completed online questionnaires assessing physical appearance perfectionism, fear of negative evaluation, and depressive symptoms using validated scales. Data were analyzed to examine a moderated mediation model.

**Results:**

Our findings reveal a significant positive correlation between physical appearance perfectionism and subthreshold depression (*r* = 0.48, *p* < 0.001). Fear of negative evaluation emerged as a significant mediator in this relationship (*t* = 5.553, 95% *CI* = [0.084, 0.194]). Notably, gender moderated the association between appearance perfectionism and fear of negative evaluation, with female students demonstrating a stronger relationship between these variables.

**Findings:**

These results underscore the detrimental effects of appearance-related perfectionism in the context of Chinese cultural norms. The study highlights the importance of addressing both physical appearance perfectionism and fear of negative evaluation in mental health interventions for college students. Educational institutions, families, and society should implement strategies to promote healthy body image perceptions and mitigate the impact of negative evaluation fears. Furthermore, our findings emphasize the need to consider gender-specific approaches, as social expectations and gender role differentiation appear to influence the psychological mechanisms underlying subthreshold depression.

## 1 Introduction

Modern societies place a high value on perfect physical appearance. The halo effect reinforces the belief that a ‘perfect appearance’ ensures success, happiness, love, popularity, and career advancement (1). Media platforms, including magazines, TV, the internet, and social media, constantly promote idealized bodies. Physical appearance significantly influences social interactions and information exchange. For instance, attractiveness often determines interactions with the opposite sex (2). University students, at a critical stage of identity development, are highly sensitive to these societal narratives. This sensitivity is closely linked to physical appearance perfectionism (PAP). Physical appearance perfectionism focuses on achieving flawless looks (3). Perfectionism is a personality trait with three types: self-oriented, other-oriented, and socially prescribed (4). PAP involves striving for flawlessness and setting unrealistically high appearance standards. It includes harsh self-criticism (5), fear of negative evaluations (6), and intense social pressure (7). College students face strong societal pressures to conform to gendered beauty ideals. Both men and women often strive for perfection and feel dissatisfied with their appearance (8). Recent studies show that appearance perfectionists use makeup, photo editing, and even plastic surgery to enhance their looks. These behaviors reflect the gap between ideal and real appearance. They also highlight concerns about imperfections and fear of negative evaluations (9).

Fear of negative evaluation (FNE) is defined in the DSM-5 (Criterion B). It refers to the fear of being judged negatively by others (10). Individuals with high FNE experience more social stress in evaluative situations. Prolonged stress can worsen physical and mental health (11). FNE is a core cognitive bias leading to maladaptive responses in social and performance settings (12). It is strongly linked to low self-esteem, low well-being, and social avoidance. These factors significantly influence subthreshold depression (13–16). FNE can also increase suicidal ideation, a key feature of depression (17, 18).

Subthreshold depression (StD) is highly prevalent among college students (40.8%) (19). This rate is much higher than in the general population (11). StD is also called minor, mild, or subclinical depression (20). It is marked by depressive moods and various behavioral, somatic, and pathological symptoms (21). StD reduces quality of life, impairs social functioning, and increases the risk of major depression (22). Despite its high vulnerability, StD is often overlooked. It does not meet clinical diagnostic criteria for depression but can rapidly progress to depressive disorders. It is also associated with high rates of self-inflicted suicidality (23). The exact causes of StD remain unknown. In this study, a score of ≥16 on standardized scales identifies StD.

Physical appearance perfectionism is strongly linked to gender. Studies show gender differences in appearance-related concerns. Men tend to focus on gaining weight and height. Women tend to prioritize weight loss and slenderness (1). Objectification theory suggests women pay more attention to observable attributes like appearance and body size. They often view themselves from an external perspective (24). Using a cutoff of ≥16 for StD, this study explores the mechanisms linking appearance perfectionism to SD. The findings have implications for early intervention in public mental health.

The social disengagement model links socially prescribed perfectionism to depression (25). This connection may impact both physical and mental health. Few studies have examined physical appearance perfectionism in college students. This study aims to fill this gap. It investigates the relationships between PAP, FNE, and StD. It also explores the mechanisms of StD formation and the negative effects of PAP.

## 2 Materials and methodology

### 2.1 Measures

#### 2.1.1 Participants and procedure

Participants were recruited via convenience sampling through an online questionnaire distributed between late April and June 2023 in Henan and Shandong Provinces, China. Specifically, data were collected from students at Zhoukou Normal University and Binzhou Medical University. Classes were selected non-randomly based on accessibility through teacher contacts, thus not meeting strict random sampling criteria.

Inclusion criteria for the questionnaire were: (1) university students aged 17–28 years; (2) voluntary participation with signed informed consent. Exclusion criteria included: (1) completion time < 120 s (indicating potential non-serious responses); (2) StD scale scores <16; (3) self-reported or clinically diagnosed mental disorders.

This study adhered to ethical guidelines and was approved by the Ethics Committee of Qingdao Mental Health Center (Approval No. 2023013). Participants were informed that the survey was solely for research purposes, with guaranteed privacy protection. They retained the right to participate or withdraw at any stage (26).

#### 2.1.2 Physical appearance perfectionism (PAP)

The Physical Appearance Perfectionism Scale (PAPS), developed by Yang and Stoeber (3), assesses individual differences in perfectionism related to physical appearance. It comprises two dimensions: hope for perfection and worry about imperfection. These dimensions exhibit distinct relationships with social adaptation: worry about imperfection is more strongly associated with maladaptive evaluative concerns, while hope for perfection aligns with positive goal striving (1). Using the Chinese version of PAPS, we measured hope for perfection (5 items, e.g., “I want my body form to be perfect”) and worry about imperfection (7 items, e.g., “I worry that my appearance is not good enough”). Responses were recorded on a 5-point Likert scale (1 = not at all; 5 = very much), with total scores ranging from 12 to 60. Higher scores indicate stronger physical appearance perfectionism. The scale demonstrated high reliability (Cronbach’s *α* = 0.874 for the full scale, 0.818 for hope for perfection, and 0.849 for worry about imperfection). Confirmatory factor analysis (CFA) indicated acceptable model fit (*χ*^2^/df = 5.318, GFI = 0.945, IFI = 0.937, CFI = 0.937, TLI = 0.922, RMSEA = 0.073), supporting the scale’s validity.

#### 2.1.3 The center for epidemiological studies depression scale (CES-D)

The CES-D, originally developed by Radloff and revised by Ren et al. (27), was used to assess depressive symptoms over the past week. The 20-item scale includes three dimensions: somatic symptoms, depressed mood, and positive evaluation. Items are scored on a 4-point Likert scale (0 = not at all; 3 = all the time), with the positive evaluation dimension reverse-scored. Total scores range from 0 to 60, with higher scores indicating more severe depression; scores >16 indicate subthreshold depression. In this study, the scale showed high reliability (Cronbach’s *α* = 0.884). CFA results confirmed acceptable model fit (*χ*^2^/df = 3.960, GFI = 0.924, IFI = 0.923, CFI = 0.923, TLI = 0.912, RMSEA = 0.060), supporting its validity.

#### 2.1.4 Fear of negative evaluation scale (FNE-S)

The fear of negative evaluation scale (Chinese version), adapted by Cao and Qi (28) from Lear’s original, measures individuals’ fear of external negative evaluations. The 12-item scale includes 8 positively and 4 negatively worded items, scored on a 5-point Likert scale (1 = not at all; 5 = all). Higher scores indicate greater fear of negative evaluation. Based on Lin’s (29) findings, only the positively scored items were used in this study. The scale demonstrated high reliability (Cronbach’s *α* = 0.862). CFA results indicated acceptable model fit (*χ*^2^/df = 7.430, GFI = 0.958, IFI = 0.945, CFI = 0.937, TLI = 0.923, RMSEA = 0.089), supporting its validity.

### 2.2 Data analysis

Data were analyzed using SPSS 25.0. Common method bias was assessed using Harman’s single-factor test. Normality of continuous variables was evaluated through skewness and kurtosis tests. Additional analyses included the Durbin-Watson test, homogeneity of variance, Bartlett’s test of sphericity, multicollinearity tests, descriptive statistics, inter-group differences, partial correlations, independent samples t-tests, and ANOVA. Mediation and moderated mediation models were tested using Process v3.5 (Model 4 and Model 7, respectively), with bias-corrected percentile bootstrap methods (5,000 resamples, α = 0.05). Covariates were controlled, and all variables were standardized.

#### 2.2.1 Common method bias and normality test

Self-reported data may introduce common method bias. Harman’s single-factor test revealed five factors with eigenvalues >1, with the largest factor explaining 30.87% of variance (<40% threshold), indicating no significant bias (26). Normality tests showed skewness (−0.472 to 0.943) and kurtosis (−0.027 to −0.475) values within acceptable ranges (<3), confirming normal distribution.

#### 2.2.2 Durbin–Watson test and homogeneity of variance

The Durbin–Watson test (value = 0.454) assessed data independence. Homogeneity of variance was confirmed using the White test (*p* = 1.00), indicating no significant heteroscedasticity.

#### 2.2.3 Bartlett’s test of sphericity and collinearity test

Bartlett’s test of sphericity (*p* < 0.01) confirmed significant correlations among variables, suggesting shared underlying factors. Multicollinearity tests revealed variance inflation factors (VIFs) ranging from 1.077 to 1.955 (<3) and tolerance values between 0.511 and 0.929 (>0.1), indicating no multicollinearity issues (30).

## 3 Results

### 3.1 Statistical methods

#### 3.1.1 Descriptive statistics and inter-group differences in gender

A total of 1,114 students were surveyed, with 820 valid participants (293 males, 527 females) retained after applying strict exclusion criteria. The average age was 20.78 years (SD = 2.04; range = 17–28). Demographic characteristics included: 36.70% male, 64.30% female; 43.50% urban, 56.50% rural; 26.60% freshmen, 28.70% sophomores, 22.70% juniors, 11.60% seniors, and 10.50% postgraduate students. Age distribution: 0.37% aged 17, 10.00% aged 18, 18.54% aged 19, 24.15% aged 20, 16.59% aged 21, 12.07% aged 22, and 18.29% aged 23–28. Only-child status: 27.30% only children, 72.70% non-only children.

Means and standard deviations were computed, and gender and grade differences were analyzed (Table 1). No significant gender differences were found in worry about imperfections, but significant differences emerged in hope for perfection, PAP, FNE, and StD, with males scoring higher than females (*p* < 0.05). Grade differences were observed only in StD, with seniors and juniors showing higher prevalence than freshmen, sophomores, and graduate students [*F*(4,820), *p* = 0.02].

**Table 1:** Descriptive statistics and univariate statistics for research measures.

Main variables	Gender	M ± SD	*t*	Cohen’s *d*
PAP	Men	42.28 ± 0.556	5.19***	**0.378**
	Women	38.94 ± 0.366		
Hope for perfection	Men	28.13 ± 0.379	0.939	0.068
	Women	26.36 ± 0.259		
Worry about imperfection	Men	33.84 ± 0.679	7.172***	**0.377**
	Women	25.46 ± 0.322		
FNE	Men	23.77 ± 0.371	3.955***	0.145
	Women	20.72 ± 0.24		
StD	Men	18.51 ± 0.248	12.589***	**0.768**
	Women	18.22 ± 0.183		

***P < 0.001 (two-tailed). PAP, physical appearance perfectionism; FNE, fear of negative evaluation; StD, subthreshold depression (*N* = 820). The bold values indicate statistically significant effect sizes.

### 3.2 Partial correlations

Partial correlation analysis controlled for age and grade to examine relationships among key variables: gender, worry about imperfection, hope for perfection, PAP, FNE, and StD (Table 2). All main variables, except gender, were positively correlated (*p* < 0.05) (Table 3).

**Table 2:** Correlations among key variables.

	1	2	3	4	5	6
1. Gender	1					
2. Worry about imperfection	−0.228^***^	1				
3. Hope for perfection	−0.035	0.523^***^	1			
4. PAP	−0.169^***^	0.915^**^	0.822^***^	1		
5. FNE	−0.146^***^	0.646^***^	0.535^***^	0.684^***^	1	
6. StD	−0.367^***^	0.555^***^	0.248^***^	0.48^***^	0.451^***^	1
*M*		21.81	18.33	40.14	26.99	28.46
SD		6.01	4.21	8.95	6.20	9.97

**P < 0.01, ***P < 0.001 (two-tailed). PAP, physical appearance perfectionism; FNE, fear of negative evaluation; StD, subthreshold depression (*N* = 820).

**Table 3:** Analysis of mediation effects.

	Effect	SE	*t*	LLCI	ULCI
Total effect	0.432	0.029	15.007^***^	0.376	0.489
Direct effect	0.293	0.038	7.612^***^	0.217	0.368
Indirect effect	0.139	0.028	5.553^***^	0.084	0.194

***P < 0.001 (two-tailed). PAP, physical appearance perfectionism; FNE, fear of negative evaluation; StD, subthreshold depression (*N* = 820).

Bootstrap sample size = 5,000; *CI* = confidence interval. (*N* = 820).

### 3.3 Moderated mediation effects

#### 3.3.1 Mediation analysis

**Figure 1:**
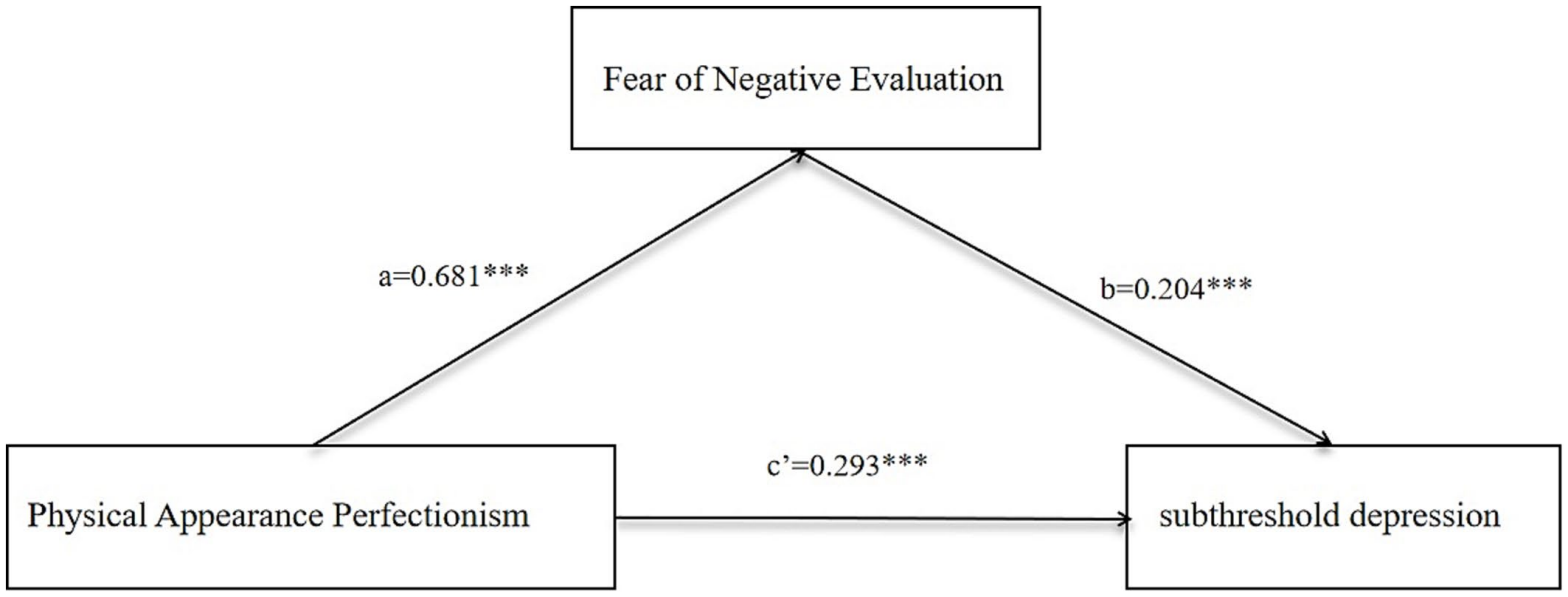
The mediating role of fear of negative evaluation. Structural equation modeling results for the relationship between physical appearance perfectionism, fear of negative evaluation, and subthreshold depression. All coefficients in the figures are standardized and significant at the 0.05 level. The numerical values on the paths represent the key standardized coefficients (β) from the regression analysis. ***P < 0.001.

Variables were standardized (*Z*-scores), and additional covariates (age, gender) were controlled. The SPSS PROCESS macro was used with 5,000 bootstrap samples to test mediation effects. Fear of negative evaluation mediated the relationship between physical appearance perfectionism and subthreshold depression (*β* = 0.204, SE = 0.038, *t* = 5.353, *p* < 0.001; 95% *CI* [0.084, 0.194]). Physical appearance perfectionism positively predicted subthreshold depression (*β* = 0.432, SE = 0.029, *t* = 15.007, *p* < 0.001), even after accounting for fear of negative evaluation (*β* = 0.293, SE = 0.038, *t* = 7.612, *p* < 0.001) (Figure 1).

#### 3.3.2 Moderated mediation analysis

**Figure 2:**
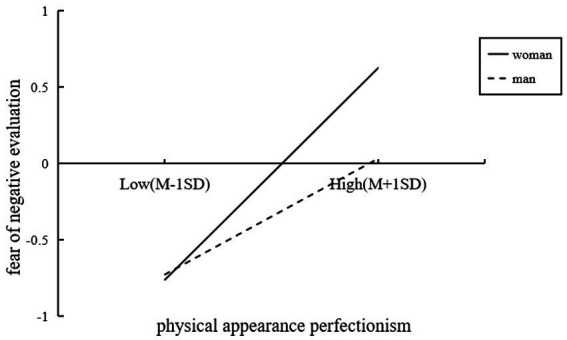
The simple slope test about gender evaluation. The moderating of gender in the relationship between physical appearance perfectionism and fear of negative evaluation. *X*-axis: physical appearance perfectionism; *Y*-axis: fear of negative evaluation. Two line represent men and women, respectively. With an increase in the level of physical appearance perfectionism, the fear of negative evaluation rises among both men and women. However, the rate of increase is significantly steeper for women compared to men, suggesting that women are more vulnerable to experiencing fear of negative evaluation as a consequence of heightened physical appearance perfectionism (*β* = −0.132, SE = 0.053, *t* = 2.490, *p* < 0.05).

Model 7 revealed gender moderated the mediation pathway. Physical appearance perfectionism positively predicted fear of negative evaluation (*β* = 0.675, SE = 0.026, *t* = 25.885, *p* < 0.001), and the interaction term (perfectionism × gender) was significant (*β* = −0.132, SE = 0.053, *t* = −2.490, *p* < 0.05; 95% *CI* [−0.236, −0.028]). Women exhibited stronger sensitivity to fear of negative evaluation with increasing perfectionism (Figure 2).

## 4 Discussion

This cross-sectional study examined the link between physical appearance perfectionism and subthreshold depression, mediated by fear of negative evaluation and moderated by gender. Results aligned with prior research, showing no gender differences in hope for perfection (31) but higher scores among males in other variables. Social expectations and the halo effect may explain these differences, as men are frequently bestowed with greater social expectations to exhibit a masculine, strong, neat, and charming image. To a certain extent, such image requirements drive men to pay increased attention to their appearance grooming in order to conform to social image settings such as that of a “successful man,” which also reflects the halo effect. The halo effect is a cognitive bias that confirms individuals’ tendency to infer overall personality traits, such as ability and morality, from a single prominent characteristic, such as appearance (32). For example, Dion et al. (33). Through experiments, it has been proved that people with high attractiveness are automatically given higher social expectations, such as a 15% increase in intelligence and a 23% increase in social ability. And this effect has been verified in cross-cultural research. In addition, the competitive pressure in modern society is on the rise. In an attempt to stand out in the competition of marriage and dating, men may place greater emphasis on the perfection of their own appearance and seek to enhance their competitiveness through a favorable external image.

Results showed a positive correlation between physical appearance perfectionism, fear of negative evaluation, and subthreshold depression, and fear of negative evaluation mediated the relationship between perfectionism and subthreshold depression. Specifically high appearance perfectionism may amplify an individual’s negative appraisal fear and thereby exacerbate subthreshold depression, consistent with social adaptation account of depression theory. Subthreshold depression occurs when expectations exceed what an individual is capable of achieving when the high standards and demands of the ideal do not match or even fall far short of real-life levels (34). According to Maslow’s Hierarchy of Needs Theory, everyone has the need to improve themselves, strive for progress, make a good impression on others and stay away from making negative impressions, particularly perfectionists, when it comes to their appearance (35). The cognitive theory of depression also maintains that negative evaluations from others might confirm an individual’s cognitive bias pattern of failure, thereby resulting in depressive emotions. In this study, fear of negative evaluation was a psychological state of excessive worry and fear, and it may be associated with some cognitive biases and then lead to depression.

Gender moderated this pathway, with women showing heightened sensitivity to negative evaluations, likely due to societal objectification (36) and physiological differences in emotional processing. It holds that appearance and body shape are the most significant relation of a woman, and that patriarchal societies have always used, bought, and sold women as sexual commodities. In terms of appearance perfectionism, girls bear more pressure from people around them to evaluate their appearance, and they are more afraid of some external negative evaluation (37). Furthermore, neurobiological and psychological differences between genders likely contribute to this phenomenon: Females exhibit greater emotional sensitivity and a stronger tendency toward rumination—a cognitive pattern linked to sustained negative affect and delayed emotional recovery. Compounding these factors, cyclical fluctuations in estrogen levels have been associated with increased neuroendocrine susceptibility to depressive symptoms in females (38). This multifactorial interplay may explain the protracted stress response and attenuated emotional regulation capacity observed in female populations compared to males. Namely, under the same degree of appearance perfectionism inclination, women are more likely to experience a higher level of fear regarding negative evaluation.

This study reveals potential negative effects of influence on subthreshold depression among college students in the Chinese cultural environment. Focusing on and addressing college students’ fear of negative evaluations is critical to overcoming subthreshold depression. Here are some specific suggestions and policies: Schools should conduct mental health education courses to guide students to correctly understand their own appearance and the evaluations of others, improving their psychological adjustment abilities. Also, schools are supposed to organize diversified campus activities so that students can feel in these activities that their value does not solely depend on physical appearance but is more related to personal abilities, moral character and other factors. Finally, psychological counseling services should be provided to help students adjust their cognition and relieve psychological pressure. Society should regulate the content disseminated by the media. Relevant departments should strengthen the supervision of the media, requiring the media to advocate healthy and natural aesthetic concepts in their dissemination. Together, we should create an inclusive social atmosphere, that is, build a relatively relaxed and inclusive social environment.

### 4.1 Limitations

This study has several limitations that warrant consideration. First, the study did not separate the dimensions of appearance perfectionism (hope for perfection vs. fear of imperfection), which may have distinct psychological impacts. Second, the sample, drawn from specific university types, may lack generalizability. Future studies should include diverse institutions and control for academic backgrounds. Additionally, the use of cross-sectional design precludes causal inferences. Longitudinal or experimental designs are needed. Finally, only fear of negative evaluation was examined as a mediator. Additional factors like self-esteem and face concerns warrant investigation.

## 5 Conclusion

This study innovatively explored the psychological risks of physical appearance perfectionism, particularly its role in subthreshold depression through fear of negative evaluation and included men in the study. Gender moderates this relationship, with women more vulnerable to negative evaluations. These findings underscore the need for targeted interventions to address appearance-related pressures and promote mental health among college students.

## Data Availability

The raw data supporting the conclusions of this article will be made available by the authors, without undue reservation.
