# Supplementary material for: The relationship between physical appearance perfectionism on subthreshold depression in college students: the role of gender and fear of negative evaluation
Source: Front Public Health. 2025 Mar 26;13:1559815. doi: 10.3389/fpubh.2025.1559815 (PMC11978643; doi:10.3389/fpubh.2025.1559815)
Supplement: Supplementary file 1 [file Data_Sheet_1.pdf]

the complete measurement scales utilized in this study

PAPS
Worry About Imperfection (WAI)
I am not satisfied with my appearance.
I am never happy with my appearance no matter how I dress.
I worry that my appearance is not good enough.
I wish I could completely change my appearance.
My appearance is far from my expectations.
I worry about others' being critical of my appearance.
I often think about shortcomings of my appearance.

Hope For Perfection (HFP)
I hope my body shape is perfect.
I hope that I look attractive.
I hope others admire my appearance.
I hope others find me attractive.
I hope I am handsome/beautiful.

BFNES
1.I worry about what other people will think of me evenwhen I know it doesn't make any difference.
2.I am frequently afraid of other people noticing my shortcomings.
3.I am afraid that others will not approve of me.
4.I am afraid that people will find fault with me.
5.When I am talking to someone, I worry about what they may be thinking about me.
6.I am usually worried about what kind of impression I make.
7.Sometimes I think I am too concerned with what other people think of me.
8.I often worry that I will say or do the wrong things.

CED-S
I was bothered by things usually don't bother me
2 My appetite was poor
3 I felt that I could not shake off the blues even with help from my family or friends
4 I felt I was just as good as others
5 I had trouble keeping my mind on what I was doing
6 I felt depressed
7 I felt that everything I did was an effort
8 I felt hopeful about the future
9 I though my life had been a failure
10 I was fearful
11 My sleep was restless
12 I was happy
13 I talked less than usual
14 I felt lonely
15 People were unfriendly
16 I enjoyed life
17 I had crying spells
18 I felt sad
19 I felt that people disliked me
20 I could not get "going"
